# A Novel Trichomonas vaginalis Surface Protein Modulates Parasite Attachment via Protein:Host Cell Proteoglycan Interaction

**DOI:** 10.1128/mBio.03374-20

**Published:** 2021-02-09

**Authors:** Brenda M. Molgora, Anand Kumar Rai, Michael J. Sweredoski, Annie Moradian, Sonja Hess, Patricia J. Johnson

**Affiliations:** a Molecular Biology Institute, University of California, Los Angeles, Los Angeles, California, USA; b Department of Microbiology, Immunology, & Molecular Genetics, University of California, Los Angeles, Los Angeles, California, USA; c Proteome Exploration Laboratory, Division of Biology and Biological Engineering, Beckman Institute, California Institute of Technology, Pasadena, California, USA; University of Georgia

**Keywords:** *Trichomonas vaginalis*, adherence, proteomics, glycosaminoglycans, heparan sulfate, host-pathogen interactions

## Abstract

Trichomonas vaginalis is a highly prevalent, sexually transmitted parasite which adheres to mucosal epithelial cells to colonize the human urogenital tract. Despite adherence being crucial for this extracellular parasite to thrive within the host, relatively little is known about the mechanisms or key molecules involved in this process. Here, we have identified and characterized a T. vaginalis hypothetical protein, TVAG_157210 (TvAD1), as a surface protein that plays an integral role in parasite adherence to the host. Quantitative proteomics revealed TvAD1 to be ∼4-fold more abundant in parasites selected for increased adherence (MA parasites) than the isogenic parental (P) parasite line. *De novo* modeling suggested that TvAD1 binds *N*-acetylglucosamine (GlcNAc), a sugar comprising host glycosaminoglycans (GAGs). Adherence assays utilizing GAG-deficient cell lines determined that host GAGs, primarily heparan sulfate (HS), mediate adherence of MA parasites to host cells. TvAD1 knockout (KO) parasites, generated using CRISPR-Cas9, were found to be significantly reduced in host cell adherence, a phenotype that is rescued by overexpression of TvAD1 in KO parasites. In contrast, there was no significant difference in parasite adherence to GAG-deficient lines by KO parasites compared with wild-type, which is contrary to that observed for KO parasites overexpressing TvAD1. Isothermal titration calorimetric (ITC) analysis showed that TvAD1 binds to HS, indicating that TvAD1 mediates host cell adherence via HS interaction. In addition to characterizing the role of TvAD1 in parasite adherence, these studies reveal a role for host GAG molecules in T. vaginalis adherence.

## Abstract (precis)

The ability of the sexually transmitted parasite Trichomonas vaginalis to adhere to its human host is critical for establishing and maintaining an infection. Yet how parasites adhere to host cells is poorly understood.

## INTRODUCTION

Trichomonas vaginalis is a flagellated, eukaryotic parasite and the etiologic agent of trichomoniasis, the most common nonviral sexually transmitted infection worldwide. The global burden of T. vaginalis infections is high, with reports of roughly 156 million new cases worldwide and over 276 million cases annually (1, 2). In the United States, an estimated 3.7 million people are currently infected with T. vaginalis (3, 4). Although T. vaginalis infections are primarily asymptomatic, trichomoniasis symptoms can include vaginitis, prostatitis, urethritis, discharge, infertility, and adverse pregnancy outcomes, such as preterm delivery and low birth weight in infants (5–7). Additionally, T. vaginalis has been associated with increased acquisition of HIV (8–11) and increased risk of cervical and prostatic cancers (12–15). While there are serious consequences attributed to trichomoniasis, the underlying processes of T. vaginalis pathogenesis remain poorly defined.

As T. vaginalis is an extracellular organism, its adherence to host mucosal tissues is critical for parasite survival. Attachment to the urogenital epithelium of men and women by the parasite allows for the establishment and maintenance of an infection as well as nutrient acquisition from host cells (16, 17). The adherence ability of T. vaginalis to host cells *in vitro* is strain dependent, exhibiting up to a 45-fold difference in adherence ability between strains (18). *In silico* analysis of the T. vaginalis genome identified >5,100 proteins carrying 1 or more transmembrane domains and over 300 annotated proteins from across 10 protein families with a predicted contribution to colonization and parasite cytopathogenicity (19, 20). Furthermore, studies into the molecular mechanisms of T. vaginalis attachment to the host mucosal tissues suggest that parasite adherence to the host is multifaceted with many factors yet to be defined (16, 21–25).

Due to the vast number of surface proteins potentially involved in parasite adherence to the host, the surface membrane proteomes of 3 adherent and 3 lowly adherent T. vaginalis strains were compared (26). This study identified 271 putative plasma membrane proteins with 11 of them found to be significantly more abundant in the adherent strains than in the lowly adherent strains. Following this work, a number of these putative surface proteins were further characterized as parasite adherence proteins (27–29), further validating this approach. However, the ability to strictly correlate the presence and abundance of specific proteins to the adherence phenotype in this study was limited by the high level of variability exhibited by the different T. vaginalis isolates.

Here, we developed a novel selection method to isolate isogenic T. vaginalis parasites that differ in their ability to bind host cells. This method takes advantage of reported observations that T. vaginalis *in vitro* adherence to culture tubes is correlated with its ability to bind host cells, both *in vitro* and *in vivo* (30, 31). We report the identification of the surface-expressed TVAG_157210 (TvAD1) protein and characterize its role in T. vaginalis adherence to the host. TvAD1 is a parasite surface-expressed protein predicted to interact with the host surface via binding of *N*-acetylglucosamine—a component of the host glycosaminoglycan heparan sulfate (HS). We subsequently demonstrated the role of host cell HS in parasite adherence. To our knowledge, this is the first report of a T. vaginalis surface protein interacting with host glycosaminoglycans to initiate parasite adherence to the host.

## RESULTS

### Enrichment of T. vaginalis surface proteins.

**FIG 1:**
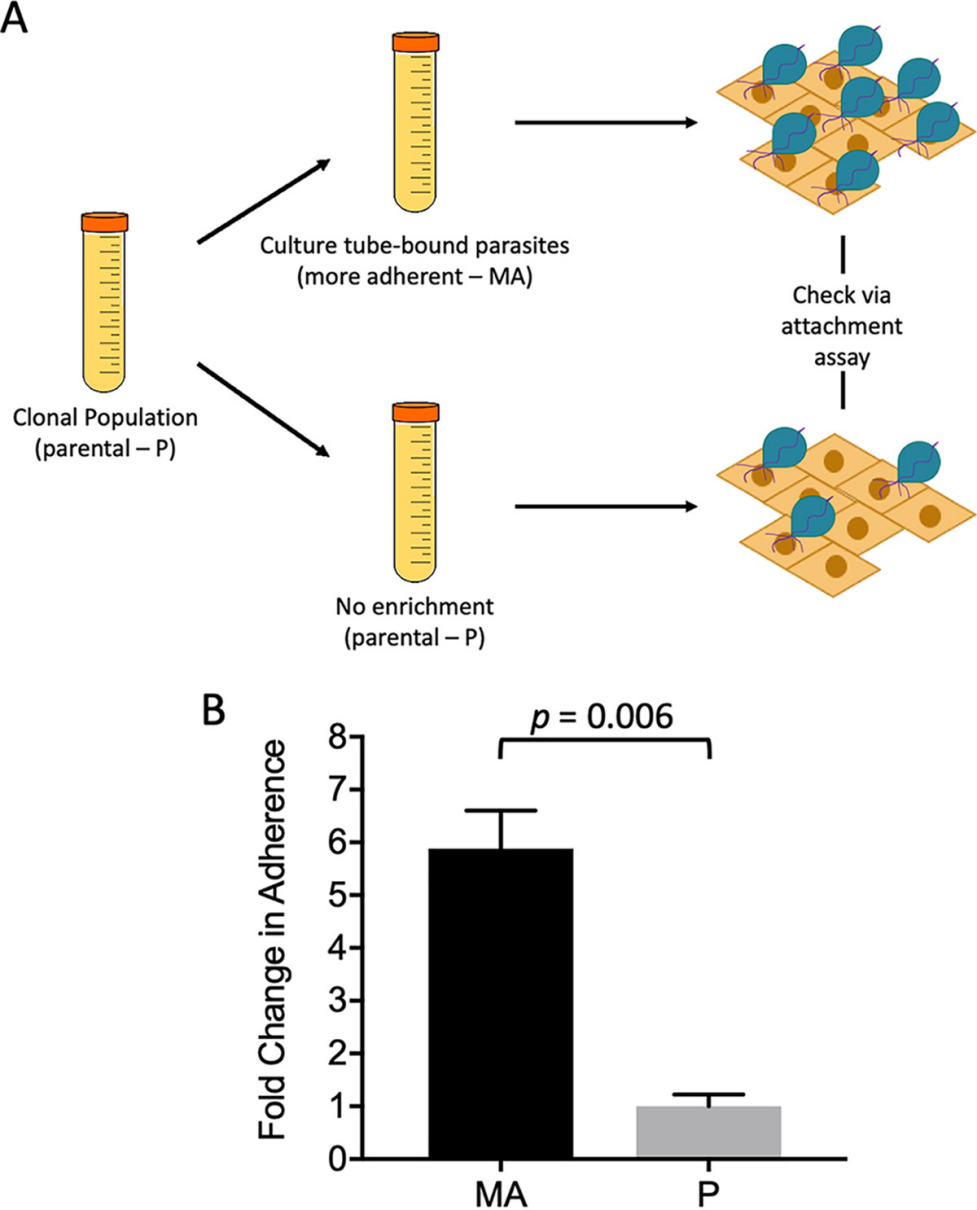
Enrichment of parasites with increased adherence to host cells. (A) Schematic for selective enrichment of T. vaginalis. Clonal population was used to generate two isogenic populations, namely, more adherent (MA) and parental (P). The MA population resulted from passaging culture tube-bound parasites daily. The P population was passaged without selection of tube-bound parasites. Changes in adherence to BPH-1 cells was measured using our standard adherence assay (18). (B) Adherence to BPH-1 cell monolayers by MA and P parasites passaged for 8 weeks. The mean of three experiments each done in triplicate is shown ± SEM. Statistical significance was determined using Student’s *t* test.

Previous analyses of the surface proteomes of 3 highly adherent and 3 lowly adherent T. vaginalis clinical isolates determined that surface proteins are differentially expressed on strains, conferring varying adherence to the host as a result. However, the highly variable expression of predicted surface proteins (26) by different T. vaginalis strains limited these analyses. To eliminate this issue, we developed a method that would allow us to study increased parasite adherence to host cells using an isogenic, clonal strain of T. vaginalis. Taking advantage of the observation that the parasite’s *in vitro* adherence to culture tubes is correlated with its ability to bind host cells, both *in vitro* and *in vivo* (30, 31), we selected parasites from the isogenic culture by passaging only parasites bound to the tubes (Fig. 1A). After 8 weeks of daily passaging of only culture tube-adherent parasites, we obtained an isogenic strain, called more adherent (MA). The original parental (P) strain was also passaged daily for 8 weeks without selection. Adherence assays were then done to quantify and compare the adherence of MA (see Fig. S1A in the supplemental material) and P (Fig. S1B) strains to benign prostate hyperplasia 1 (BPH-1) cells. We found that our selection approach significantly increased adherence of MA to host cells by approximately 6-fold compared with that of P (Fig. 1B). These isogenic parasite populations that differ in their adherence to host cells set the stage for identifying membrane proteins that are more abundant in MA parasites and hence possibly involved in parasite adherence to the host.

10.1128/mBio.03374-20.1 FIG S1 Labeled LSU 160 MA parasites are greater in number per field of view/image than P. LSU 160 MA (A) and P (B) parasites were labeled with CellTracker Red and subjected to an attachment assay using BPH-1 monolayers as described in the Materials and Methods. Average number of parasites per field/image for MA and P was 83 and 11, respectively. Representative images for both isogenic strains are shown. Download FIG S1, DOCX file, 0.07 MB. Copyright © 2021 Molgora et al. 2021 Molgora et al. This content is distributed under the terms of the Creative Commons Attribution 4.0 International license.

### TMT multiplex proteomic analysis identified differences in the abundance of proteins between MA and P parasites.

Our laboratory and others have successfully used biotinylation of surface proteins to obtain enriched surface protein fractions for use in proteomic studies (26, 32, 33). Using membrane-impermeable sulfo-NHS-SS-biotin (sulfosuccinimidyl-2-[biotinamido]ethyl-1,3-dithiopropionate), the surface membrane proteins of both MA and P populations were labeled. Protein labeling was confirmed by indirect immunofluorescence assay (IFA) using 488-conjugated streptavidin. Staining was confined to the surface and shows minimal cytosolic staining (see Fig. S2A in the supplemental material), demonstrating that the surface proteins were selectively biotinylated. In addition, the membrane-enriched fractions from biotinylated and control nonbiotinylated samples were compared by SDS-PAGE and streptavidin-horseradish peroxidase (HRP) Western blot. Similar protein profiles were seen between the samples following SDS-PAGE separation (Fig. S2B, left panel) confirming equivalent extraction between samples. As expected, only the biotinylated sample showed signal on the streptavidin-HRP Western blot (Fig. S2B, right panel), confirming that surface proteins were biotinylated and that the likelihood of proteins in the control samples binding streptavidin-conjugated proteins is quite low.

10.1128/mBio.03374-20.2 FIG S2 T. vaginalis surface proteins were biotinylated using sulfo-NHS-SS-biotin. (A) Parasite surface proteins were biotinylated using sulfo-NHS-SS-biotin whose sulfonate group inhibits it from permeating the parasite membrane. Immunofluorescence microscopy of biotinylated parasites reacted with streptavidin conjugated to a 488 fluorophore is shown for MA. P strain had comparable staining. (B) SDS-PAGE analysis of 10 μg of membrane-enriched fractions prior to protein purification by streptavidin. Biotin-labeled (B) and unlabeled proteins (NB) were separated by SDS-PAGE and silver stained (left panel) or detected by Western blotting with streptavidin-HRP-conjugated antibody (right panel). Molecular weight markers are shown on the left of each panel. Download FIG S2, PDF file, 0.2 MB. Copyright © 2021 Molgora et al. 2021 Molgora et al. This content is distributed under the terms of the Creative Commons Attribution 4.0 International license.

**FIG 2:**
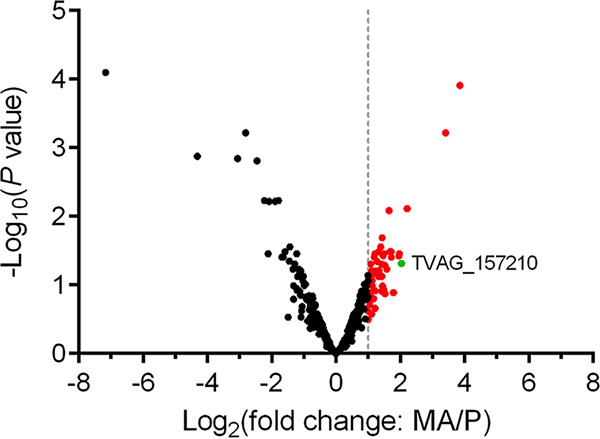
Multiplex proteomics illustrate differentially abundant proteins in MA compared with those in P. A total of 365 proteins were identified by proteomics analyses of MA and P, in triplicate (see Table S1). Volcano plot of −log_10_(adjusted *P* value) is plotted against log_2_(fold change: MA/P) as measured by TMT multiplexing and LC-MS/MS. The non-axial vertical line denotes ≥2-fold change protein abundance of MA versus P. Red points correspond to proteins 2-fold or higher in abundance MA/P. The single green point corresponds to TvAD1 (TVAG_157210). Statistical analysis was performed for triplicate samples by two-sided *t* test.

The parasite membrane and the subsequent biotinylated proteins were then fractionated via centrifugation using freeze-thaw cell lysis followed by sonication. This membrane-enriched fraction was detergent-solubilized to release the proteins and was passed through a streptavidin affinity column to enrich for biotinylated proteins. To identify surface proteins and their relative abundance levels on MA and P, three biologically independent surface-enriched samples from each were labeled with tandem mass tag (TMT) isobaric labels for downstream quantitative analysis using liquid chromatography-tandem mass spectrometry (LC-MS/MS) (34, 35). Analysis of protein identifications obtained from the MA and P samples identified 365 total T. vaginalis proteins (see Table S1 in the supplemental material). Of these proteins, 22% were predicted to be membrane proteins based on the previously published surface membrane proteome and analysis of the T. vaginalis genome (19, 26). To graphically represent the quantitative data, a volcano plot of −log_10_(*P* value) versus log_2_(fold change: MA/P) was constructed (Fig. 2). Data points to the right of the right-most non-axial vertical line, colored red, denote proteins which exhibited fold changes of MA/P greater than 2. After excluding contaminating proteins which include abundant hydrogenosomal and ribosomal proteins that are common contaminants of subcellular fractions and have been found to contaminate all subcellular fractions of T. vaginalis that we have subjected to proteomic analyses (26, 29, 36), 28 proteins identified by this multiplexed proteomics approach were predicted to be membrane or membrane-associated proteins with a 2-fold or greater abundance in MA than in P (Table 1). Several proteins were found to be less abundant in MA than in P by 2-fold or more; the gene number and description of these proteins are listed in Table S2 in the supplemental material.

**TABLE 1:** Differentially expressed predicted membrane or membrane-associated proteins^*a*^

			Protein features		
Locus	Description	Fold change	TMD^*b*^	MAP^*c*^	Reference
TVAG_293660	Conserved hypothetical protein	14.47			26
TVAG_477640; TVAG_335250	Conserved hypothetical protein	10.63	Yes		26, 29
TVAG_228160; TVAG_150940; TVAG_203740	Coactosin	4.62		Yes	
TVAG_539120	Conserved hypothetical protein	4.30			26
TVAG_157210	Conserved hypothetical protein	4.09	Yes		26
TVAG_226630	Conserved hypothetical protein	3.93			
TVAG_464410	AMP dependent ligase/synthetase	3.86			
TVAG_222040	4-α-Glucanotransferase	3.45			26
TVAG_407150; TVAG_419690	arp2/3 Complex 16-kD subunit	3.20		Yes	
TVAG_270790	*N*-Acylglucosamine-2-epimerase	2.87			
TVAG_369030	Clathrin heavy chain	2.86		Yes	
TVAG_270770	Conserved hypothetical protein	2.81			
TVAG_283380	FMR1-interacting protein	2.69			
TVAG_321740	Conserved hypothetical protein	2.67	Yes		26
TVAG_369020; TVAG_558650; TVAG_516070; TVAG_502180	Clathrin heavy chain	2.54		Yes	26
TVAG_348080	Calcium-transporting ATPase	2.50	Yes		
TVAG_180570	FERM domain protein	2.49		Yes	
TVAG_151920; TVAG_429360; TVAG_145570	WD repeat domain protein	2.37			
TVAG_376130	Gelosin	2.32		Yes	
TVAG_028160	Cation-transporting ATPase	2.31	Yes		26
TVAG_000810	Conserved hypothetical protein	2.30			
TVAG_147050	Conserved hypothetical protein	2.25	Yes		26
TVAG_258230	Conserved hypothetical protein	2.19	Yes		26
TVAG_013580	Hypothetical protein	2.16			
TVAG_059980	Conserved hypothetical protein	2.15	Yes		26
TVAG_000880	GP63-like	2.14	Yes		19, 26
TVAG_038850	Conserved hypothetical protein	2.13	Yes		26
TVAG_185680	Conserved hypothetical protein	2.00	Yes		26

a With a 2-fold or higher abundance in membrane-enriched fractions of MA parasites than that of P parasites.

b TMD, transmembrane domain.

c MAP, membrane-associated protein.

10.1128/mBio.03374-20.8 TABLE S1 Quantitative proteomic data from plasma membrane-enriched fractions of MA and P parasites. Protein identification number and annotation are as listed in the TrichDB.org database. Proteins were cross referenced with previous studies (19, 26, 29, 59). The log_2_(MA/P), fold change(MA/P), and adjusted (adj.) *P* values are provided. Positive log_2_(MA/P) values refer to an increased protein abundance in MA versus P parasites, while a negative log_2_(MA/P) value refers to an increased protein abundance in P versus MA parasites. (TMD, transmembrane domain; SP, signal peptide). Download Table S1, XLS file, 0.2 MB. Copyright © 2021 Molgora et al. 2021 Molgora et al. This content is distributed under the terms of the Creative Commons Attribution 4.0 International license.

10.1128/mBio.03374-20.9 TABLE S2 Proteins >2-fold less abundant in MA parasites than in P parasites. Locus and description are as listed in the TrichDB.org database. Fold change refers to the reduction in protein abundance in MA versus P parasites. Download Table S2, DOCX file, 0.01 MB. Copyright © 2021 Molgora et al. 2021 Molgora et al. This content is distributed under the terms of the Creative Commons Attribution 4.0 International license.

The predicted functions and gene copy number of the proteins found to have the highest increase in abundance in MA relative to P were examined. Similar multicopy genes that give rise to proteins that cannot be differentiated by the proteomic data are referred to as protein groups. The 2 top-ranked protein groups, TVAG_293660 and TVAG_335250/TVAG_477640, belong to large protein families (https://trichdb.org/trichdb/) which would complicate functional analyses. The next most abundant protein group (TVAG_ 228160, TVAG_150940, and TVAG_539120) is predicted to include cytoskeletal-associated proteins that may be in close contact with the plasma membrane. As manipulation of cytoskeletal proteins is likely to result in complex phenotypes, which may indirectly affect parasite binding, we chose not to pursue these proteins.

A hypothetical protein, without similarity to other known proteins, TVAG_157210, was the 5th most abundant protein/protein group identified, with ∼4-fold higher abundance on MA parasites. BLAST analyses (https://blast.ncbi.nlm.nih.gov/Blast.cgi) of TrichDB failed to find homologues, demonstrating that TVAG_157210 is a single-copy gene in the T. vaginalis genome. As a first step toward characterizing this protein, topological analysis using the TOPCONS consensus prediction of membrane protein topology program was used (37). These analyses indicate that TVAG_157210 contains a single transmembrane domain at the C terminus at positions 275 to 295, predicting that the bulk of the protein is exposed on the outer surface of the parasite. We then used InterPro (38) and Pfam (39) analyses which failed to detect any functional domains. Protein structure prediction software predicts TVAG_157210 to be involved in cell adhesion and/or protein binding, albeit with a low confidence interval due to the lack of suitable protein structure templates for modeling (40, 41). However, *de novo* modeling by the i-TASSER program in conjunction with the 3DLigandSite program suggested that TVAG_157210 binds *N*-acetylglucosamine (GlcNAc) (42, 43). Given that proteoglycans, of which some contain high levels of GlcNAc, are known to be displayed on the epithelial cells T. vaginalis binds to in the urogenital tract (44–46) and other pathogens are known to utilize proteoglycans for host cell attachment (47–50), we decided to validate and further investigate this protein, which we renamed Tv adherence protein 1 (TvAD1).

As a first step to validate the increased abundance of TvAD1 on MA parasites, we compared TvAD1 mRNA levels between MA and P parasites, using real-time quantitative reverse transcription-PCR (qRT-PCR). TvAD1 mRNA levels were found to be 1.82-fold (*P *= 0.0005) greater in MA than in P, consistent with increased protein levels in the MA versus P surface proteome (see Fig. S3 in the supplemental material). Furthermore, to confirm that TvAD1 alone was capable of conferring an increased adherence phenotype, TvAD1 was exogenously expressed in the poorly adherent G3 strain by nucleofecting the parasites with our standard T. vaginalis expression vector where expression of the TvAD1 gene was driven by the alpha succinyl-CoA-synthetase (αSCS) promoter (26, 51, 52). Expression of the C-terminal tagged TvAD1 protein was confirmed by anti-hemagglutinin (HA) Western blot (see Fig. S4A in the supplemental material). The adherence of parasites overexpressing TvAD1 and G3 parasites containing an empty vector (EV) were then compared. Increased expression of TvAD1 in the poorly adherent parasites significantly increased attachment to BPH-1 cells ∼2.6-fold compared with parasites nucleofected with EV (*P *= 0.007) (Fig. S4B). These data directly demonstrate that TvAD1 plays a role in T. vaginalis adherence to the host.

10.1128/mBio.03374-20.3 FIG S3 mRNA expression of TvAD1 mirrors increased protein abundance of MA strain. mRNA expression level of TvAD1 (TVAG_157210) identified as a differentially expressed protein by multiplex proteomics was examined by qRT-PCR. Data were normalized to tubulin expression levels and are expressed as fold change compared with P ± standard deviation. Statistical significance was determined for samples done in triplicate by Student’s *t* test. Download FIG S3, DOCX file, 0.04 MB. Copyright © 2021 Molgora et al. 2021 Molgora et al. This content is distributed under the terms of the Creative Commons Attribution 4.0 International license.

10.1128/mBio.03374-20.4 FIG S4 Overexpression of TvAD1 increases adherence of a poorly adherent strain of T. vaginalis to prostate cells. Exogenous expression of a C-terminal 2× HA-tagged TvAD1 protein (TvAD1-OE) in the poorly adherent G3 strain was analyzed by Western blotting (A) and attachment assay (B). (A) Expression of the HA-tagged TvAD1 protein was confirmed by anti-HA Western blot and compared with parasites transfected with empty vector (EV). Detection of endogenous Hsp-70 using a T. vaginalis anti-Hsp70 antibody was used as a loading control. Expected sizes for Hsp-70 and TvAD1-OE are ∼70 kDa and ∼37 kDa, respectively. (B) Attachment of TvAD1-OE parasites to BPH-1 cells was quantified and compared with attachment of EV parasites. Data shown are means of triplicate experiments ± SEM. Statistical significance was determined using an unpaired *t* test with Welch’s correction. Download FIG S4, DOCX file, 1.6 MB. Copyright © 2021 Molgora et al. 2021 Molgora et al. This content is distributed under the terms of the Creative Commons Attribution 4.0 International license.

### Mammalian glycosaminoglycans play a role in MA parasite adherence to epithelial cells.

**FIG 3:**
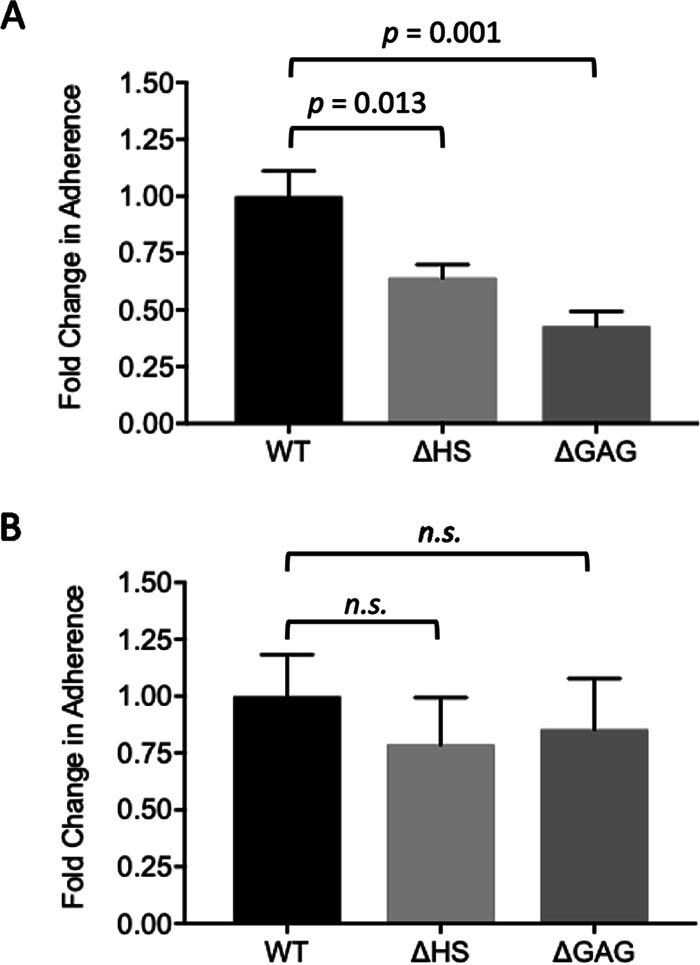
Increased adherence of MA strain mediated by host cell proteoglycans. Ability of MA (A) and P (B) parasites to adhere to wild-type (WT), heparan sulfate-deficient (ΔHS), and GAG-deficient (ΔGAG) CHO cell monolayers was quantified following a 30-min incubation. Data shown are the means ± SEM from independent experiments, with each performed in triplicate. Statistical significance was determined using a one-way analysis of variance (ANOVA) with Tukey’s multiple-comparison test.

GlcNAc belongs to a large class of amino sugars that comprise the glycosaminoglycan (GAG) heparan sulfate (HS) (51) found on epithelial cells and in the extracellular matrix (52). To determine if GAGs play a role in T. vaginalis adherence, we used Chinese hamster ovary (CHO) cell lines generated by Jeffrey Esko and colleagues that lack surface HS (ΔHS), a component of GAGs, or all GAGs (ΔGAG) (53, 54). Compared with the wild-type CHO cell line (WT), a reduction in adherence of MA parasites of 36% (*P = *0.013) and 57% (*P = *0.001) was observed using ΔHS cells and ΔGAG cells, respectively (Fig. 3A). In contrast, adherence of the isogenic P parasites was not significantly reduced on ΔHS or ΔGAG cells, showing a 21% (*P = *0.785) and 15% (*P = *0.878) reduction in adherence, respectively, compared with that of WT (Fig. 3B). These observations indicate that host glycosaminoglycans mediate adherence of MA to host cells.

### CRISPR-Cas9 gene knockout of TvAD1 significantly reduced MA parasite adherence to host cells.

**FIG 4:**
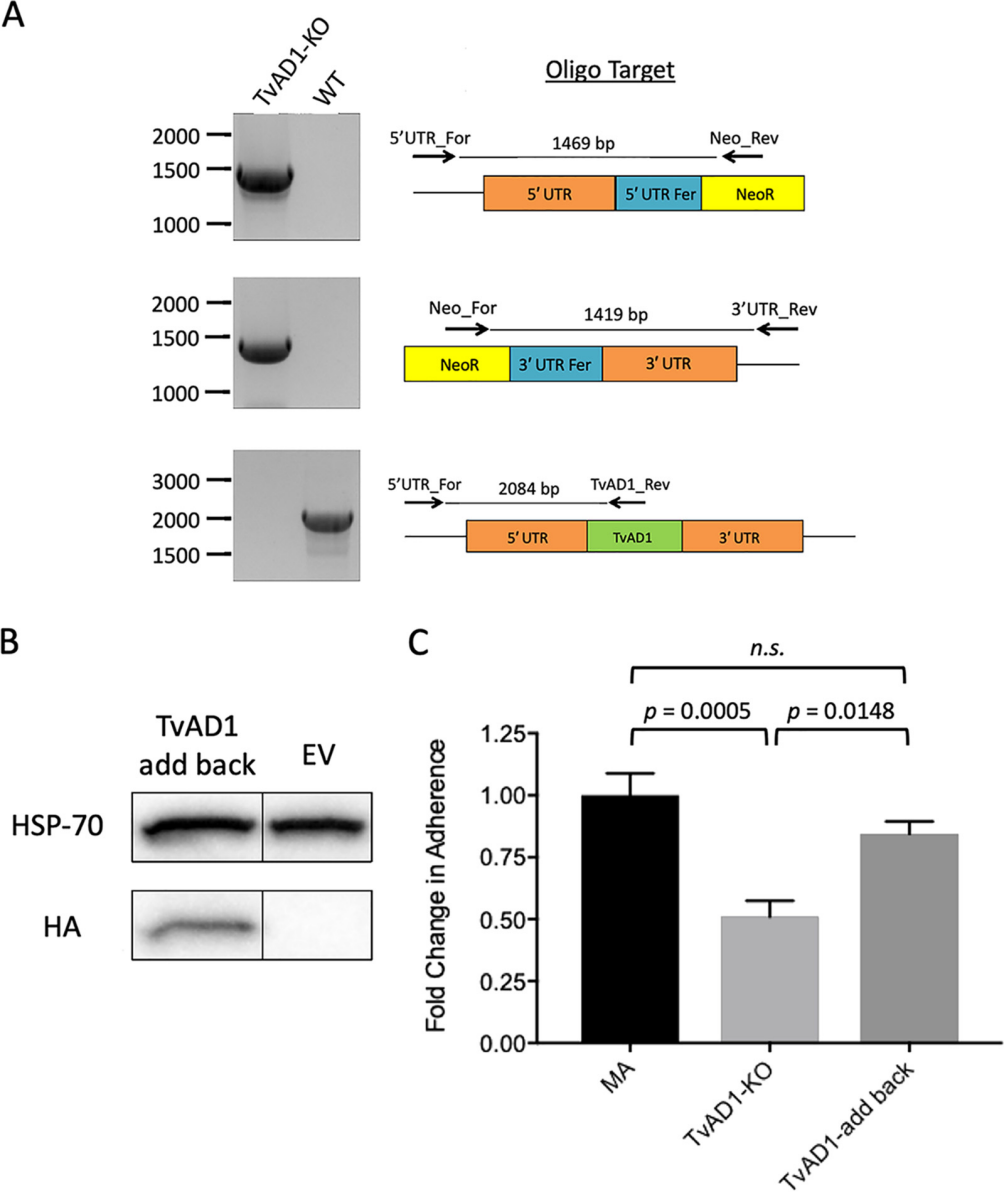
CRISPR-Cas9 knockout of TvAD1 significantly reduces adherence of MA parasites to BPH-1 cell monolayers. (A) PCR analysis of the TvAD1 knockout (TvAD1-KO) and wild-type (WT) parasites for the presence of the neo gene in the TvAD1 locus. PCRs using primers to amplify the 5′ UTR integration site (5UTR_For + Neo_Rev) yielded the expected 1,469-bp product (top panel), while primers used to amplify the 3′ UTR integration (Neo_For + 3UTR_Rev) yielded the expected 1,419-bp product (middle panel), showing that the neomycin gene is present in the TvAD1 locus. The intact TvAD1 locus product of 2,084 bp detected in WT parasites using primers located upstream of the 5′ integration site and within the TvAD1 gene (5UTR_For + 157internal_Rev (bottom panel) is absent in TvAD1-KO parasites. (B) Episomal expression of a C-terminal 2× HA-tagged TvAD1 protein in the KO background (TvAD1-add back) was confirmed by anti-HA immunoblot analyses and compared with parasites transfected with empty vector (EV). Hsp-70 was used as a loading control. Expected sizes for Hsp-70 and TvAD1-add back are ∼70 kDa and ∼37 kDa, respectively. The black line between TvAD1-add back and EV indicates the blot was spliced to remove a lane between the samples. (C) Attachment of TvAD1-KO and TvAD1-add back to BPH-1 cell monolayers was quantified and compared with the attachment of MA parasites. Data shown are means of triplicate biological replicates, which were performed in triplicate ± SEM. Statistical significance was determined using a one-way ANOVA with Tukey’s multiple-comparison test.

To further elucidate the role of TvAD1 in host cell adherence, CRISPR-Cas9 gene editing, which we recently established in T. vaginalis (55), was employed to knock out the gene. Using homology-directed repair, the TvAD1 gene in MA parasites was replaced with the neomycin resistance gene flanked by arms homologous to the 5′ and 3′ untranslated regions (UTRs) of TvAD1 to allow selection of TvAD1 gene knockout parasites. Confirmation of the loss of TvAD1 from the genome was done using three different PCRs (Fig. 4A). Integration of the knockout cassette in the correct locus was confirmed by PCRs amplifying from the 5′ UTR or 3′ UTR of the gene into the newly integrated neomycin gene. As expected, the TvAD1 knockout (TvAD1-KO) parasites present an amplicon of expected size in both the 5′ UTR + neo (top panel) and 3′ UTR + neo (middle panel) exhibiting the predicted sizes of 1,469 bp and 1,419 bp, respectively. The third PCR confirmed the absence of the TvAD1 in the genome, compared with WT MA parasites (bottom panel). These data confirm that we successfully knocked out TvAD1 in MA parasites.

Having knocked out TvAD1 from MA parasites, the TvAD1-KO parasites were then assayed for their ability to adhere to host cells. TvAD1-KO parasites exhibited a 49% reduction in adherence to BPH-1 cells compared with the MA wild-type parasites (*P* = 0.0005) (Fig. 4C). To ensure that the loss in adherence observed in TvAD1-KO was specific to the absence of TvAD1, TvAD1 levels were partially restored by exogenously expressing TvAD1 with 2× HA at the C terminus and assayed to determine if the KO phenotype was rescued. Western blot analysis using an anti-HA antibody confirmed that the TvAD1 protein was being expressed in the TvAD1-add back strain (Fig. 4B). When assayed for changes in adherence, we found that TvAD1-add back parasites exhibited a partial KO rescue at 84% adherence to BPH-1 cells compared with that of MA (*P = *0.0005) (Fig. 4C). In addition, TvAD1-add back adherence was significantly rescued compared with that of TvAD1-KO (*P* = 0.0148). Together, these results provide definitive evidence that TvAD1 plays a role in adherence of T. vaginalis to host cells.

### TvAD1 is necessary for heparan sulfate-mediated adherence of MA parasites to host cells.

**FIG 5:**
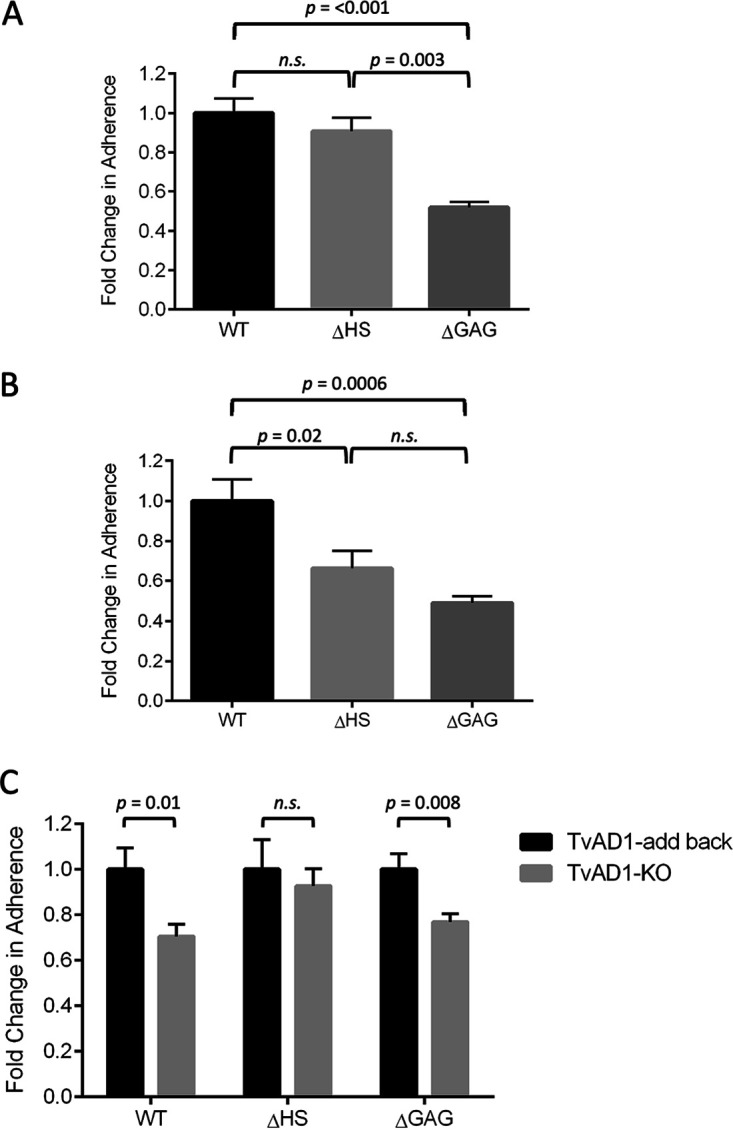
The loss of heparan sulfate on CHO cells significantly decreases adherence of TvAD1-add back parasite but not TvAD1-KO adherence. TvAD1-KO (A) and TvAD1-add back parasites exogenously expressing TvAD1 in the knockout background (B) were measured for their adherence to wild-type (WT), heparan sulfate-deficient (ΔHS), and GAG-deficient (ΔGAG) CHO cell monolayers. (C) Comparison of TvAD1-KO and TvAD1-add back parasite adherence to CHO cell monolayers. Data shown are means of independent experiments done in triplicate ± SEM. Statistical significance for A and B was determined using a one-way ANOVA with Tukey’s multiple-comparison test and unpaired *t* test for C.

With functional analyses having confirmed TvAD1 plays a role in parasite adherence to host cells and bioinformatic analyses suggesting an interaction between TvAD1 and GlcNAc, we asked whether the presence or absence of TvAD1 affects MA parasite binding to ΔHS and ΔGAG CHO cell lines by comparing binding of the TvAD1-KO and TvAD1-add back parasites. Unlike the significant decrease in adherence of MA to ΔHS cells shown in Fig. 3, we observed that adherence of TvAD1-KO parasites to ΔHS cells is not significantly decreased (*P *= 0.52) compared with WT (Fig. 5A). Adherence of TvAD1-KO to ΔGAG was significantly decreased by 48% (*P *= 0.003), consistent with data demonstrating that T. vaginalis adherence to host cells is multifactorial (21). In contrast, adding back TvAD1 to KO parasites resulted in a significant decrease in adherence of 34% (*P *= 0.02) (Fig. 5B), similar to the adherence observed for WT MA to ΔHS cells (Fig. 3A). Likewise, adherence of TvAD1-add back parasites to ΔGAG cells was significantly decreased by 51% (*P *= 0.0006) relative to adherence to WT CHO cells. Analysis of TvAD1-KO and TvAD1-add back parasite adherence to WT CHO cells revealed that adherence was significantly reduced by ∼30% (*P* = 0.01) in the absence of TvAD1 (Fig. 5C), which is similar to what was observed for these strains on BPH-1 cells (Fig. 4C). Conversely, TvAD1-KO parasites exhibited ∼7% reduction in adherence to ΔHS cells compared with TvAD1-add back (*P *= 0.62), demonstrating that episomal expression of TvAD1 conferred the increased adherence seen in the TvAD1-add back parasites and that it was mediated through an interaction with HS on the host surface. Adherence to ΔGAG by TvAD1-KO parasites exhibited ∼23% lower adherence than that of TvAD1-add back parasites (*P *= 0.008), suggesting that TvAD1 may also be interacting with other proteins on the host cell surface to mediate adherence even in the absence of all GAG molecules. These data further confirmed a role for host GAG molecules in T. vaginalis adherence to the host and strongly indicated that TvAD1-mediated adherence requires the presence of host cell heparan sulfate.

### Thermodynamic analysis determined an interaction between TvAD1 and heparan sulfate.

**FIG 6:**
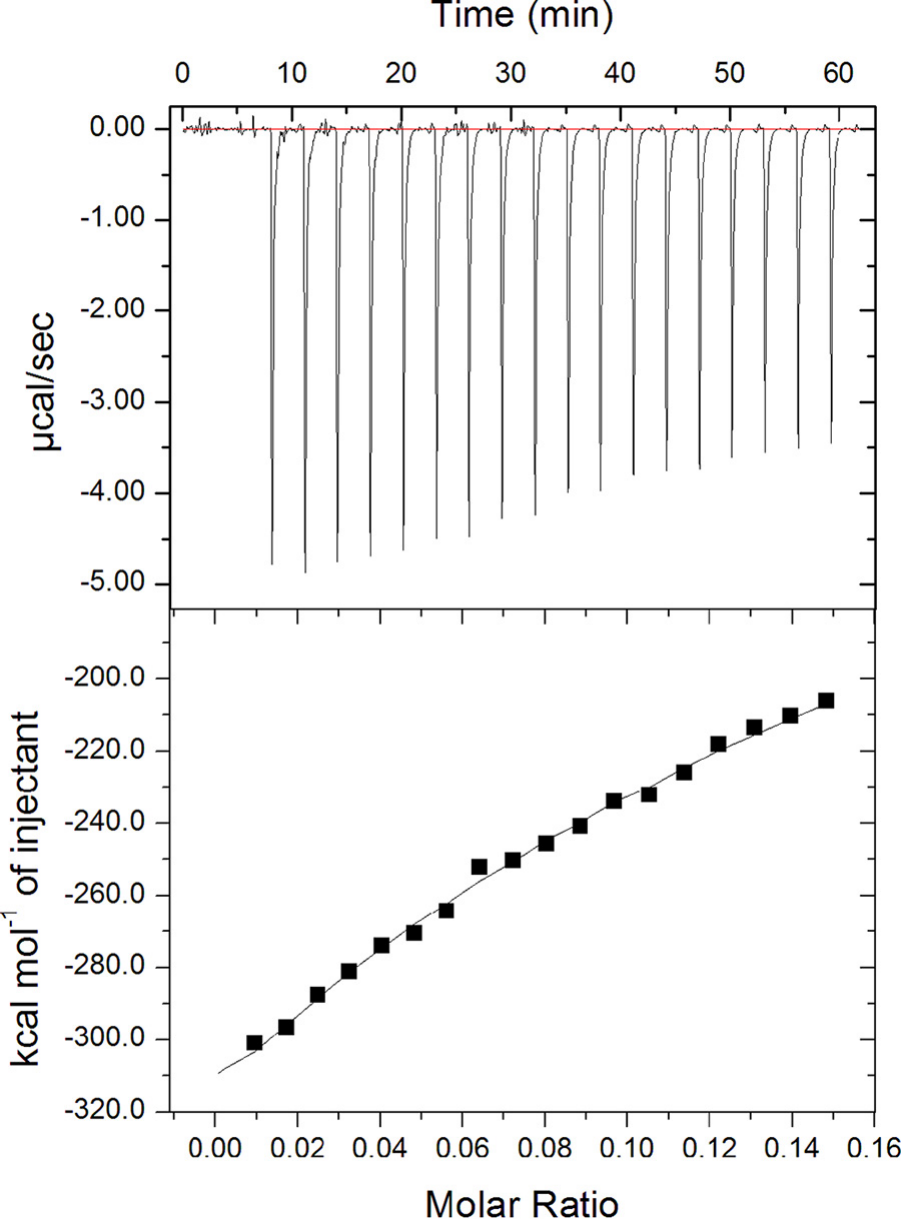
Measuring interaction between TvAD1 and heparan sulfate by ITC. Binding interaction between TvAD1 protein (146.2 μM) and heparan sulfate (HS) (1 mg/ml) was measured using ITC at 25°C. Initial injection was 0.2 μl of HS followed by 19 injections of 2 μl each applied with an interval of 3 min. The binding isotherm profile was obtained excluding the initial data point. The top panel depicts the calorimetric output during the injection of HS into the rTvAD1 solution. The bottom panel depicts the binding isotherm.

To measure whether TvAD1 interacts with HS, we employed an isothermal titration calorimeter (ITC) to measure the thermodynamics that would be associated with this interaction. Recombinant TvAD1 protein (rTvAD1), lacking its short, hydrophobic C-terminal transmembrane domain to increase solubility and protein refolding, was generated and isolated using 8 M urea under denaturing conditions. Protein purification was confirmed by SDS-PAGE and Coomassie staining (see Fig. S5 in the supplemental material). The protein was then refolded by stepwise dialysis to remove urea, and refolding was confirmed using intrinsic tryptophan fluorescence measurements. Folded rTvAD1 exhibited a peak emission wavelength (λ_max_) of 337 nm, which is well within the expected 330 -to 345-nm range for folded proteins (see Fig. S6 in the supplemental material). We tested the TvAD1-HS interaction by titrating HS onto rTvAD1 and measuring the heat change associated with this interaction. The resulting heat changes were integrated and fitted to obtain thermodynamic parameters of binding. With progressive HS injections, the thermogram, displayed by clear and distinct peaks, decreased as more TvAD1 bound HS but failed to reach saturation (Fig. 6). The heat changes were fitted accordingly to the predicted number of GlcNAc binding sites present on the protein. A two-sequential binding site model for TvAD1 was the best fit with dissociation constants (*K_d_*) of 10.57 μM and 9.62 μM, respectively. These analyses demonstrate that TvAD1 binds HS.

10.1128/mBio.03374-20.5 FIG S5 Purified His-tagged rTvAD1 obtained from Ni-NTA affinity chromatography under denaturing conditions. Eluted His-tagged rTvAD1 was analyzed by SDS-PAGE and Coomassie stain for purity. Expected rTvAD1 size is ∼35 kDa. Molecular weight markers are shown on the left. Download FIG S5, DOCX file, 1.8 MB. Copyright © 2021 Molgora et al. 2021 Molgora et al. This content is distributed under the terms of the Creative Commons Attribution 4.0 International license.

10.1128/mBio.03374-20.6 FIG S6 Intrinsic tryptophan fluorescence emission spectra of refolded and urea-denatured recombinant TvAD1 protein. Recombinant TvAD1 (rTvAD1) degree of refolding by step-wise dialysis was evaluated by measuring intrinsic tryptophan fluorescence emission. The intrinsic tryptophan fluorescence emission spectra of the protein were recorded from 300 to 450 nm upon excitation at 290 nm, and the excitation and emission slit widths were set at 5 nm. The fluorescence intensity of refolded rTvAD1 without denaturant (solid line) and under denaturing conditions (4 M urea, ■; 8 M urea, ▴) was normalized to the refolded rTvAD1 maximum fluorescence intensity. The non-axial vertical lines denote the peak emission wavelengths (λ_max_). rTvAD1 λ_max_ is 337 nm, rTvAD1 + 4 M urea λ_max_ is 345 nm, and rTvAD1 + 8 M urea λ_max_ is 362 nm. The higher λ_max_ values of urea-denatured samples fall near or within the expected 350- to 365-nm range for fully denatured proteins. Download FIG S6, DOCX file, 0.06 MB. Copyright © 2021 Molgora et al. 2021 Molgora et al. This content is distributed under the terms of the Creative Commons Attribution 4.0 International license.

## DISCUSSION

Using a novel selection method to create isogenic parasites that differ in their ability to adhere to host cells, followed by proteomic and bioinformatic analyses, we have identified and characterized a T. vaginalis surface protein, TvAD1 (TVAG_157210). We have shown that TvAD1 binds host cell glycosaminoglycans (GAGs) increasing parasite adherence to host cells. Quantitative analyses of the cell surface proteomes of more adherent (MA) and parent (P) isogenic parasites identified 29 surface proteins that are >2-fold more abundant in MA than in P parasites. Subsequent knockout of TvAD1 from MA parasites, which was found to be 4.13-fold more abundant than in P parasites, using CRISPR-Cas9, showed that the loss of TvAD1 significantly reduced the parasite’s enhanced adherence capacity. Further investigation by assaying adherence to GAG-deficient cell lines and ITC analysis identified host HS as necessary for parasite adherence by TvAD1. Previous studies using pathogenic bacteria and viruses that bind to and modify HS have established a role for GAG molecules in host colonization (47–50). However, to our knowledge, this is the first report of a T. vaginalis surface protein interacting with host cell GAG molecules and thereby enhancing parasite adherence to the host.

T. vaginalis adherence to the host epithelium is a multifaceted process in which a number of parasite factors have been shown to play a role (19, 24–29, 36, 56–58). Unlike previous proteomic analyses exploited to identify these factors, our use of isogenic strains for proteomic analysis and comparison not only allows for the identification of membrane proteins involved in adherence but also safeguards against detection of proteins that are not involved in parasite adherence but simply differ in abundance between strains.

Of the 29 membrane or membrane-associated proteins found to be >2-fold more abundant in MA parasites, 14 were identified in the previous proteome analyses as more abundant in 3 highly adherent versus 3 poorly adherent T. vaginalis strains (26), of which 2 were further characterized in additional studies (29, 59). The presence of adherence proteins, such as cadherin-like protein (26, 27) and BspA-like proteins (19, 60, 61) in our data set, further validates our approach.

A 4-α-glucanotransferase (TVAG_222040) was also found to be >3-fold more abundant in MA parasites (Table 1). This finding is notable as we recently showed that a member of this protein family found on the surface of T. vaginalis extracellular vesicles that is 88% identical to TVAG_222040 binds to HS, as well as to GAGs on the surface of host cells (59). The observation that two unrelated proteins, which bind HS and host cell GAGs, are increased in abundance of MA parasites underscores the likely importance of the interaction of T. vaginalis surface proteins and host cell proteoglycans in parasite adherence, a necessary step in colonization of the host urogenital tract.

In humans, the amino sugar *N*-acetylglucosamine (GlcNAc) is most commonly found comprising GAGs—large polysaccharides present on the proteoglycans of epithelial cells and extracellular matrix (52). Belonging to a large class of amino sugars, which serve a number of functions throughout the human body, GlcNAc is well known for its role in comprising the GAG heparan sulfate (HS) (51). We demonstrated that TvAD1 specifically binds HS and that mammalian cells lacking HS are deficient in binding MA parasites. Unlike MA, the adherence of P parasites was not significantly changed in the absence of any GAG molecule on the mammalian cell, suggesting that our novel adherence selection method selected for carbohydrate-binding factors. As the culture tubes lack a bioavailability of cellular factors, it is possible that the horse serum in our culture media provided complexes of proteins, lipids, and carbohydrates coating the culture tube to which the parasite surface molecules bind.

Analysis of the function of TvAD1 led us to employ CRISPR-Cas9 (55) to knock out the protein to directly ascertain whether it plays a role in adherence, more specifically HS-mediated adherence. Knock out of TvAD1 significantly reduced MA adherence to host cells and exogenously expressed TvAD1-add back partially restored the lost adherence phenotype. The lack of a full restoration of the adherence phenotype in the KO add back parasites is likely due to reduced levels of TvAD1 in the parasite membrane, relative to MA parasites. Notably, KO parasites showed no significant difference in adherence to HS-deficient host cells compared with the wild type. Moreover, when TvAD1 is added back to the KO parasites, a significant difference in binding to wild-type and the HS-deficient host cells reappears, thus signifying a necessary role for TvAD1 in HS-mediated adherence of MA parasites to host cells.

A direct interaction between TvAD1 and HS was shown using ITC analyses. While a two-sequential binding site model for TvAD1 is predicted as the best fit, ITC results were unable to show an affinity interaction between the two molecules, signifying that the interaction between HS and TvAD1 is not strong and may be one of avidity rather than affinity. The TvAD1 and HS interaction data suggest that TvAD1 requires the presence of additional T. vaginalis surface proteins to either initiate or stabilize adherence to host cells. Nevertheless, together, these findings provide definitive evidence that T. vaginalis binds GAG molecules for adherence to host cells and that TvAD1 plays a role in this interaction via interaction with host HS molecules.

Whether other proteins or protein families involved in parasite adherence interact with host GAG molecules has yet to be determined. With the goal of possibly designing therapeutic targets to inhibit the establishment of infection, a better understanding of the types and importance of T. vaginalis adherence factors will be necessary.

## MATERIALS AND METHODS

### Parasites, cell culture, and media.

T. vaginalis strains LSU 160 and G3 (ATCC PRA-98; Kent, UK) were cultured in Diamond’s modified Trypticase-yeast extract-maltose (TYM) medium supplemented with 10% horse serum (Sigma-Aldrich), 10 U/ml penicillin and 10 μg/ml streptomycin (Gibco), 180 μM ferrous ammonium sulfate, and 28 μM sulfosalicylic acid (62, 63). Parasites were grown at 37°C and passaged daily. Human benign prostate hyperplasia 1 (BPH-1) epithelial cells were cultured in RPMI 1640, l-glutamine, and HEPES media (Gibco) supplemented with 10 U/ml penicillin, 10 μg/ml streptomycin, and 10% fetal bovine serum (FBS; Gibco) as previously described (64). K1 wild-type Chinese hamster ovary (CHO) cell line was obtained from ATCC (CCL-61). CHO proteoglycan mutant D677 (heparan sulfate defective, ΔHS) and A745-23A1 (GAG deficient, ΔGAG) cells (53, 54) were a gift from Jeffrey D. Esko, University of California San Diego, La Jolla, CA. CHO cell lines were cultured in F12 medium (Gibco) supplemented with 10% FBS and 10 U/ml penicillin and 10 μg/ml streptomycin. BPH-1 and CHO lines were grown at 37°C with 5% CO_2_.

### Parasite selection for increased adherence.

A clonal population of T. vaginalis strain LSU 160 was derived using limiting dilution cloning. To select for the more adherent (MA) parasite, this clonal population of parental (P) parasites was cultured for ∼24 hours, after which all free-floating, unbound parasites were discarded. The culture tube was then filled with fresh completed Diamond’s media, incubated on ice for 10 min, and vortexed for 30 sec to release bound parasites. These parasites were counted by hemocytometer and passaged at a dilution of 5 × 10^4^ cells/ml into 50 ml fresh Diamond’s media. This selection process was carried out daily for 8 weeks. P parasites were passaged daily as previously described (18). Briefly, overnight cultures of P parasites were placed on ice for 10 min and then vortexed for 30 sec before being passaged at a dilution of 5 × 10^4^ cells/ml into 50 ml fresh Diamond’s media.

### Attachment assay.

Attachment of T. vaginalis parasites to BPH-1 was performed as previously described (18). Briefly, epithelial cells were seeded on 12-mm coverslips in 24-well plates at 1.75 × 10^5^ cells/well in culture medium and grown to confluence at 37°C with 5% CO_2_ for 2 days. Coverslips were washed with fresh complete RPMI medium prior to the addition of parasites. T. vaginalis was labeled with 10 mM CellTracker red CMTPX dye (Invitrogen), and 10^5^ labeled parasites were added to the monolayers in triplicate. Plates were incubated at 37°C in 5% CO_2_ for 30 min. The coverslips were washed in phosphate-buffered saline (PBS) to remove unbound parasites, fixed in 4% formaldehyde in PBS, and mounted on slides using Mowiol (Calbiochem). Fifteen images were taken per coverslip with three coverslips per condition using an Axioskop 2 epifluorescence microscope (Zeiss). Cell counts were quantified using Zen (Zeiss) and ImageJ (65) software. The scoring and counting of all images were performed in a blind fashion. Attachment of T. vaginalis parasites to CHO cells was performed as previously described with one modification—complete F12 medium was used as the culture medium. All attachment assay data are normalized and shown as fold change in adherence ± SEM. All non-normalized data are provided in Fig. S7 in the supplemental material and are shown as the average number of parasites per coverslip ± SEM from triplicate experiments.

10.1128/mBio.03374-20.7 FIG S7 Non-normalized data of all attachment assay data shown in this study. The non-normalized attachment assay data for Fig. 1B (A), Fig. 3A (B), Fig. 3B (C), Fig. 4 (D), Fig. 5A (E), Fig. 5B (F), and Fig. S4 (G) are shown as the average number of parasites per coverslip ± SEM from triplicate experiments. Download FIG S7, DOCX file, 0.4 MB. Copyright © 2021 Molgora et al. 2021 Molgora et al. This content is distributed under the terms of the Creative Commons Attribution 4.0 International license.

### Biotinylation of surface membrane proteins and purification from membrane fractions.

Biotinylation of T. vaginalis cell surface proteins was performed as described previously (26) with modifications. Briefly, 2 × 10^8^ parasites were collected and washed twice with prechilled PBS-sucrose (PBS-S; 5% sucrose) and then incubated with 0.5 mg/ml EZ-Link sulfo-NHS-SS-biotin (Thermo Scientific) in PBS-S on ice for 45 min. The reaction was quenched by adding 50 mM Tris-HCl (pH 7.4) and incubating on ice for 15 min. Biotinylation of the parasite membrane was confirmed by immunofluorescence assay using a streptavidin-488-conjugated antibody as previously described (26) and streptavidin-HRP Western blot. Biotinylated parasites were then washed three times with prechilled PBS-S and subjected to freeze-thawing. Clarification of the homogenate by centrifugation (14,000 rpm for 30 min at 4°C) was carried out to reduce cytosolic background. The membrane-enriched pellet was solubilized in 0.5 ml of lysis buffer (50 mM Tris-HCl [pH 8], 5 mM EDTA, 150 mM NaCl, 0.1% deoxycholate, 1% dodecylmaltoside, and HALT protease inhibitor), subjected to 3 sonication cycles (5-sec sonication, 30-sec recovery on ice), and incubated at 4°C for 16 to 18 h on a rotating mixer to solubilize the protein out of the membrane fraction. Streptavidin Sepharose high-performance slurry (150 μl/mg total proteins; GE Healthcare Life Sciences) was equilibrated by five washes in lysis buffer, and binding of biotinylated proteins was allowed to proceed overnight on a rotating mixer at 4°C. The resin was washed once with each of the following sterile buffers using 7× bead volume: A (6 M urea, 1% dodecylmaltoside, 1% deoxycholate, 150 mM NaCl, and 100 mM Tris-HCl [pH 8]), B (6 M urea, 0.1% dodecylmaltoside, 0.1% deoxycholate, 500 mM NaCl, 100 mM Tris-HCl [pH 8], 3.7% EtOH, and 3.7% isopropanol), and C (6 M urea and 100 mM Tris-HCl [pH 8]). After the final wash, the resin was resuspended in 50 mM tris(2-carboxyethyl)phosphine hydrochloride (TCEP) in 100 mM Tris-HCl (pH 8) and incubated at room temperature for 1 h on a rotating mixer in the dark to cleave off the biotin from the protein sample. Capture of biotinylated proteins was checked by SDS-PAGE and Western blot analysis using streptavidin-HRP (Thermo Scientific). Protein samples were snap-frozen and lyophilized.

### Proteolytic digestion and TMT 10plex labeling.

Lyophilized protein samples were proteolytically digested in solution as previously described with modifications (35, 66). Protein samples were resuspended in 8 M urea in 100 mM Tris-HCl (pH 8.5) and reduced with 500 mM TCEP for 20 min. Reduced cysteines were subsequently alkylated with 500 mM iodoacetamide for 15 min at room temperature (RT) in the dark. A total of 0.1 μg/μl Lys-C endopeptidase (Fujifilm Wako Chemicals, USA) was then added and the samples incubated at RT in the dark for 4 h to initiate proteolysis. The samples were then diluted using 100 mM Tris-HCl (pH 8.5) to a final concentration of 2 M urea and adjusted to 1 mM CaCl_2_. To generate peptides, 0.56 μg/μl mass spectrometry-grade trypsin (Promega WI) was added, and the samples were then incubated at RT in the dark for 16 to 18 h. Protein digestion was quenched by the addition of formic acid to a final concentration of 5% and lyophilized. Lyophilized protein samples were resuspended in 100 mM triethylammonium bicarbonate (TEAB) to eliminate the presence of primary amines and then labeled with TMT 10-plex labeling, according to the manufacturer’s protocol (Thermo Scientific). The pooled samples were desalted by high-performance liquid chromatography (HPLC) using an Optimize Technologies C_8_ microtrap cartridge. The desalted samples were lyophilized and then resuspended in 0.2% formic acid, which made them ready for LC-MS/MS analysis.

### Mass spectrometry analysis.

Desalted peptides were analyzed using a 26-cm analytical HPLC column (75-μm inner diameter) packed in-house with ReproSil-Pur C_18AQ_ 1.9-μm resin (120-Å pore size; Maisch, Ammerbuch, Germany). After being loaded, the peptides were separated with a 120-min gradient at a flow rate of 200 nl/min at 50°C (column heater) using the following gradient: 2% to 6% solvent B (7.5 min), 6% to 25% B (82.5 min), 25% to 40% B (30 min), 40% to 100% B (1 min), and 100% B (9 min), where solvent A was 97.8% H_2_O, 2% acetonitrile (ACN), and 0.2% formic acid and solvent B was 19.8% H_2_O, 80% ACN, and 0.2% formic acid. The Orbitrap Fusion instrument (Thermo Scientific) was operated in data-dependent acquisition mode with SPS-MS3 to automatically switch between an MS1 scan (*m/z* = 400 to 1,500) in the Orbitrap (120,000 resolution), an MS2 scan using collision-induced dissociation (CID) fragmentation and detection in the ion trap (with turbo scan rate), and an SPS-MS3 scan using higher-energy C-trap dissociation (HCD) fragmentation (65 normalized collision energy [NCE] on the top 10 most intense MS2 ions) and detection in the Orbitrap (60,000 resolution). The automatic gain control (AGC) targets of the MS1, MS2, and MS3 scans were 4E5, 1E4, and 1E5, respectively. Monoisotopic precursor selection was enabled, as well as charge state filtering (only charge states 2 to 7, ignoring undetermined charge states), minimum intensity threshold of 5,000, and dynamic exclusion of 60 seconds.

Thermo raw files were searched using MaxQuant (v. 1.5.5.1) (67, 68). Spectra were searched against UniProt T. vaginalis sequences (50,190 entries) and a contaminant database, including proteins like trypsin and human keratins (246 entries). A decoy database of reversed sequences was also included to estimate the false discovery rate. Trypsin was the specified digestion enzyme, and up to two missed cleavages were allowed. Methionine oxidation and protein N-terminal acetylation were specified as variable modifications. Carbamidomethylation of cysteine and TMT10plex modification of peptide N terminus and lysine were specified as fixed modifications. Precursor mass tolerance was 4.5 ppm after mass recalibration, MS2 ion mass tolerance was 0.5 Da, and MS3 ion mass tolerance was 0.003 Da. Score thresholds were set to achieve a 1% false discovery rate at the protein, peptide, and peptide-spectrum match levels. Calculation of iBAQ values was enabled. Proteins were further analyzed using limma (69), where a moderated *t* test was performed between protein abundances in different sample types. *P* values were adjusted using the Benjamini and Hochberg method.

### Bioinformatic analyses.

Topology of the TVAG_157210 protein was determined using TOPCONS (37). To predict TVAG_157210 function, we used Phyre2 (40), I-TASSER (42), 3DLigandSite (43), InterPro (38), Pfam (39), and PredictProtein (41) programs to analyze protein sequences and generate a protein structure *de novo*. The gene and protein sequences of TVAG_157210 were also analyzed via NCBI BLAST (https://blast.ncbi.nlm.nih.gov/Blast.cgi) to compare them against all sequences published on TrichDB (https://trichdb.org/trichdb/) as well as other organisms to search for homologues.

### Real-time quantitative reverse transcription-PCR.

A total of 2 × 10^7^ LSU160 MA and P parasites were resuspended in TRIzol to collect RNA using the Direct-zol RNA MiniPrep Plus kit (Zymo Research) following the manufacturer’s protocol. Total RNA was treated with TURBO DNA-free amplification grade DNase I (Invitrogen) and reverse transcribed using SuperScript III reverse transcriptase and oligo(dT) primers (Invitrogen). Real-time PCRs were performed using Platinum SYBR green qPCR SuperMix-UDG following the manufacturer’s protocol (Invitrogen). T. vaginalis β-tubulin was used as the housekeeping gene control. The primers for β-tubulin were Tub-For (5′-GGCTCGTAACACATCCTACTTC-3′) and Tub-Rev (5′-CTGTTGTGTTGCCGATGAATG-3′). The primers for TvAD1 were TvAD1_qPCR-For (5′-TGTTGGTGGCCTTCCAGTTTG-3′) and TvAD1_qPCR-Rev (5′-TCTGAGCAGCAGCACTTCTTG-3′). Primer specificity was checked using NCBI Primer-BLAST which indicated the primer pairs unique to the T. vaginalis sequences (70).

### CRISPR-Cas9-mediated knockout of TvAD1.

CRISPR-directed knockout of TVAD1 was performed as previously described (55) with modifications. For construction of the pCas9-2xgRNA construct targeting the TvAD1 gene, each individual gRNA cassette containing the U6 seed region and gRNA scaffold was constructed by megaprimer amplification of the gRNA scaffold. The TvAD1 gRNA-1 was constructed using primers 157gRNA1_for (5′-GTCAAACATATTCTATTACATCATCAACACTCATTCTGTTTTAGAGCTAGAAATAGC-3′) and U6-KpnI_Rev (5′-CTGCATGGTACCAAAAAATGGGACCTATCCAGA-3′), and the resulting product was purified and used in a second PCR with primer U6-SacI_For (5′-ATCTGCGAGCTCATTAAGGGTGAATGGCTAC-3′). For TvAD1 gRNA-2, the seed region was generated using primers 157gRNA2_for (5′-GTCAAACATATTCTATTACCCATTAAGACATCCTTTCGTTTTAGAGCTAGAAATAGC-3′) and U6-SacI_Rev (5′-CTGCATGAGCTCAAAAAATGGGACCTATCCAGA-3′), and its product was purified for use in a second PCR with primer U6-KpnI_For (5′-ATCTGCGGTACCATTAAGGGTGAATGGCTAC-3′). The two TvAD1-gRNA PCR products were digested with KpnI, ligated together, and purified. The resulting dual gRNA product was then digested with SacI and ligated into a derivative of pMPAC::*fkbp-cas9-gRNA* (55) that harbors no selection marker to create pMΔ::*fkbp-cas9-gRNA(TvAD1*). Construction of the TvAD1 KO cassette utilized 1,000 bp upstream of the TvAD1 start codon (5′ UTR) and 1,000 bp downstream of the stop codon (3′ UTR). PCR amplification of the 1,000-bp 5′ UTR sequence utilized primers 5UTR-157_For (5′-ATCTGCGGATCCACATGATTAATCAAAGCTATATCGATG-3′) and 5UTR-157_Rev (5′-CTGCATGGCGCGCCTAATAAAAATGAAGAGATATTTAGC-3′). PCR amplification of the 1,000-bp 3′ UTR sequence utilized primers 3UTR-157_For (5′-ATCTGCGGTACCATAAAGTAAAAGATCTTTTTTTATGTAATTTTCACAG-3′) and 3UTR_Rev (5′-CTGCATGAGCTCATATACCGAGATTTTTTTATCTATTTTCAG-3′). The 5′ UTR and 3′ UTR sequences were ligated to the NeoR gene to create the TvAD1-KO cassette. Generation of the linearized KO cassette was done using PCR and the 5UTR-157_For and 3UTR_Rev primers.

Knockout of the TvAD1 gene in the T. vaginalis LSU160 MA strain was done under nucleofection conditions as previously described (55), except the X-001 and V-kit buffer (Lonza) were utilized along with pMΔ::*fkbp-cas9-gRNA (TvAD1*) and 100 μg of linearized TvAD1-KO cassette. Immediately following nucleofection, parasites were recovered in completed Diamond’s media for 24 hours and then selected for resistance to 100 μg/ml of G418 (Gibco). After an additional 24 hours for selection, the MA parasites were pelleted, resuspended in fresh complete Diamond’s media, and redosed with 100 μg/ml G418. Drug-selected parasites were then subpopulated into 5 cells/well in a 24-well plate. When the parasites reached ∼1 × 10^7^ parasites/ml, genomic DNA was prepared for PCR screening. Initial screening was done at the 5′ UTR integration site using primer 5UTR_For (5′-GAATTCCATGTTTCAGACTGCC-3′) located upstream of the 1,000-bp 5′ UTR homology arm sequence and Neo_Rev (5′-AGCCGATTGTCTGTTGTGCCC-3′). A subpopulation with the correct 5′ UTR Neo integration was cloned via limiting dilution and rescreened for integration at the 5′ UTR using the primers above as well as integration at the 3′ UTR end using primers Neo_For (5′-CGCTATCAGGACATAGCGTTGGC-3′) and 3UTR_Rev (5′-GATCTTAACTTTGGTTACATACAAGCTG-3′), of which the latter is located downstream of the 1,000-bp 3′ UTR homology arm sequence. Screening for the wild-type TvAD1 gene used primers 5UTR_For and 157internal_Rev (5′-TCTGAGCAGCAGCACTTCTTG-3′). All PCR products were confirmed by DNA sequencing.

### Plasmid construction for TvAD1 exogenous expression in T. vaginalis parasites.

For exogenous expression of TvAD1 in poorly adherent T. vaginalis G3 parasites, TvAD1 was PCR amplified from LSU 160 MA genomic DNA using primers TvAD1_NdeI-For (5′-CATATGTTTGGACTTCTTGGACTCTCA-3′) and TvAD1_Kpni-Rev (5′-GGTACCTTATACCTTGTCTGAGCAGCAGC-3′). The resulting PCR fragment was cloned into the MasterNeo-(HA)_2_ plasmid (71). Nucleofection of the poorly adherent T. vaginalis G3 strain with 50 μg pMNeo_TvAD1-2xHA or 50 μg pMNeo_EV (empty vector) was done as described above except for the use of T cell buffer(Lonza) and the U-033 nucleofection program. Transfectants were selected and maintained using 100 μg/ml G418. When the parasites reached ∼1 × 10^7^ parasites/ml, 5 × 10^6^ parasites were taken and lysed in lysis buffer (50 mM Tris-HCl, 5 mM EDTA, 150 mM NaCl, 0.1% Nonidet P-40, 0.5% deoxycholate, 2% SDS, and HALT protease inhibitor). Protein concentrations were quantified by Bradford assay (Bio-Rad), and 10 μg protein was used to confirm expression of exogenous TvAD1 by anti-HA Western blot.

Plasmid construction for TvAD1 complementation in KO parasites was carried out in a similar fashion with the following modifications. The pMNeo_TvAD1-2xHA plasmid was digested to replace the neomycin phosphotransferase (neo) selectable marker with the puromycin *N*-acetyltransferase gene (72) as previously described (29) to generate pMPAC_TvAD1-2xHA. Nucleofection of the TvAD1-KO parasites with 50 μg pMPAC_TvAD1-2xHA or 50 μg pMPAC_EV was done as described above for the KO parasites, and transfectants were selected and maintained using 60 μg/ml puromycin dihydrochloride (A.G. Scientific, Inc.). Exogenous protein expression was confirmed by anti-HA Western blot as described above.

### Production and purification of rTvAD1.

The TvAD1 protein without the C-terminal transmembrane domain (missing residues 281 to 310) was PCR amplified from LSU 160 genomic DNA using the following primer pair: SalI157-for (5′-ATCTGCGTCGACATGTTTGGACTTCTTGGACTC-3′) and NotI157-rev (5′-ATC-TGCGCGGCCGCAACCCAAGCCCAAACTGG-3′). PCR amplicons were cloned into the pET28b(+) expression vector containing a 6×-His tag and transformed into BL21(DE3) Escherichia coli (Thermo Scientific). An overnight culture was inoculated into 1 liter LB medium supplemented with 100 mg/ml ampicillin. When the culture reached an optical density at 600 nm (OD_600_) of 0.5 to 0.6, expression was induced with 1 mM isopropyl β-d-1-thiogalactopyranoside (IPTG) for 5 h at 37°C. The culture was then spun at 5,000 × *g* for 30 min at 4°C, and the pellet was resuspended in 1 g/3 ml lysis buffer (10 mM Tris-HCl, 100 mM NaCl, 8 M urea, and HALT protease inhibitor [pH 8]).

Lysates underwent 6 cycles of freeze-thaw lysis and then were centrifuged at 22,095 × *g* for 2 h at 4°C. The clarified lysate was filtered using a 0.44-μm filter and diluted 1:3 using dilution buffer (10 mM Tris-HCl, 300 mM NaCl, and 4 M urea [pH 8]). The denatured proteins were then purified by Ni-nitrilotriacetic acid (NTA) agarose (Qiagen) affinity chromatography. rTvAD1 purity following affinity chromatography was confirmed by SDS-PAGE and Coomassie blue staining. Purified rTvAD1 was quantified by Bradford assay, diluted to 100 μg/ml using dilution buffer, and dialyzed in the same buffer overnight at 4°C to remove imidazole and reduce urea concentration to 4 M. Urea concentration was further reduced in a stepwise reduction manner (73) by dialyzing against 20 mM Tris-HCl [pH 8], 150 mM NaCl, 0.2 M l-arginine (74), 10% glycerol, and decreasing concentrations of urea at every stage (3 M, 2 M, 1.5 M, 1 M, 0.5 M, and 0.25 M). In the final step, the protein was dialyzed against 20 mM Tris-HCl [pH 8], 150 mM NaCl, 50 mM l-arginine, 50 mM l-glutamic acid (75), and 10% glycerol. Each dialysis step was carried out for a minimum of 5 h with a buffer change at 1.5 h and 3 h. Refolded protein was concentrated using sucrose reverse dialysis (76), and concentration was determined by Bradford assay.

### Intrinsic tryptophan fluorescence.

Intrinsic tryptophan fluorescence spectra of refolded rTvAD1 was recorded using a PTI QuantaMaster spectrofluorometer (Horiba) upon excitation at 290 nm. Tryptophan fluorescence emission spectra were recorded between 300 and 450 nm. The excitation and emission slits were both set at 5 nm. Integration time was set at 10 sec. All experiments were performed at 25°C using a protein concentration of 285.7 nM. The fluorescence emission spectra of refolded rTvAD1 without denaturant and under denaturing conditions (4 M and 8 M urea) were normalized to the refolded rTvAD1 maximum fluorescence intensity following subtraction of the blank buffer emission spectra values.

### ITC analysis.

Interaction of TvAD1 with heparan sulfate (HS) was monitored by isothermal titration calorimetry (ITC)-based experiments using an iTC200 instrument (MicroCal/GE Healthcare, Piscataway, NJ). All the protein samples were dialyzed in a buffer containing 20 mM Tris-HCl [pH 8], 150 mM NaCl, 50 mM l-arginine, 50 mM l-glutamic acid, and 10% glycerol. For the ITC measurement, the sample cell (cell volume of 0.250 ml) was filled with rTvAD1 (146.2 μM) and the reference cell was filled with same buffer in which the protein was dialyzed. rTvAD1 was titrated with HS using the following protocol: an initial 0.2-μl injection followed by 19 injections of 2 μl each with an interval of 3 min and under constant stirring at 750 rpm at 25°C. The binding isotherm profile was obtained omitting the initial data point. The data were fitted using Origin 7 software. The dissociation constant (*K_d_*) was determined from 1/Ka where Ka is the binding constant.
