# Supplementary figures and images for: A Novel Trichomonas vaginalis Surface Protein Modulates Parasite Attachment via Protein:Host Cell Proteoglycan Interaction
Source: mBio. 2021 Feb 9;12(1):e03374-20. doi: 10.1128/mBio.03374-20 (PMC7885099; doi:10.1128/mBio.03374-20)

A

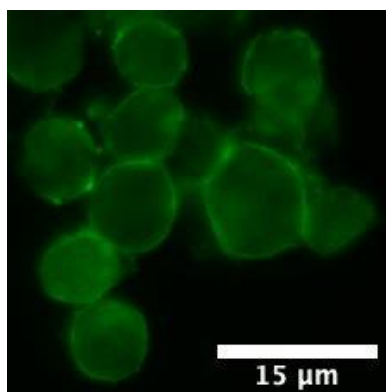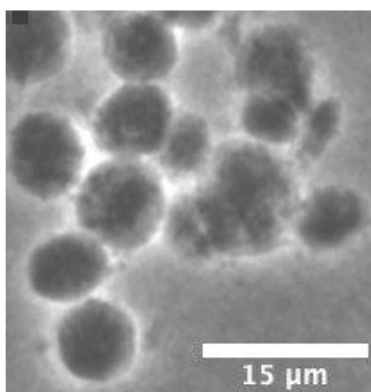

B

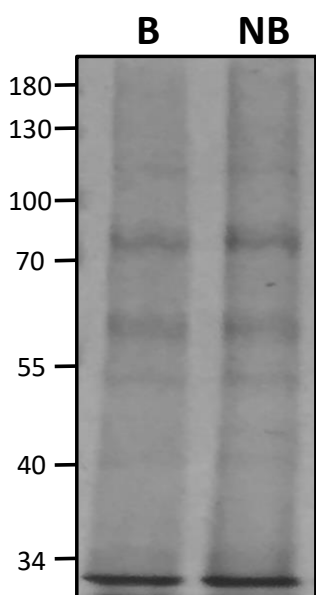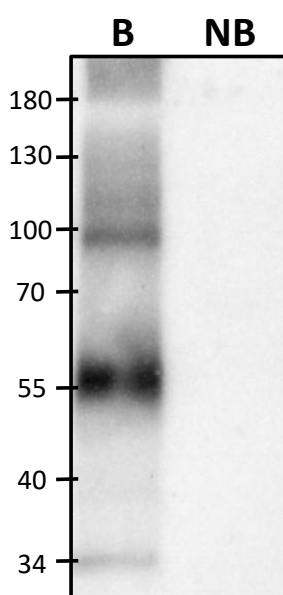

Fig. S2

Supplement: FIG S2 [file mBio.03374-20-sf002.pdf]
