# Supplementary material for: A Novel Trichomonas vaginalis Surface Protein Modulates Parasite Attachment via Protein:Host Cell Proteoglycan Interaction
Source: mBio. 2021 Feb 9;12(1):e03374-20. doi: 10.1128/mBio.03374-20 (PMC7885099; doi:10.1128/mBio.03374-20)
Supplement: TABLE S2 [file mBio.03374-20-st002.docx]

**Table S2. Proteins >2-fold less abundant in MA parasites, relative to P parasites.** Locus and description are as listed in the TrichDB database. Fold change refers to the reduction in protein abundance in MA versus P parasites.

| **Locus** | **Description** | **Fold Change** | **TMD** |
| --- | --- | --- | --- |
| TVAG_230400 | Conserved Hypothetical Protein | 143.99 | yes |
| TVAG_544880 | Conserved Hypothetical Protein | 19.93 | yes |
| TVAG_310150 | Conserved Hypothetical Protein | 5.49 | --- |
| TVAG_226870; TVAG_484340 | Conserved Hypothetical Protein | 4.68 | --- |
| TVAG_439460 | AP-2 complex subunit beta-1 | 4.24 | --- |
| TVAG_485120 | Conserved Hypothetical Protein | 3.74 | yes |
| TVAG_412220 | Malic enzyme | 3.45 | --- |
| TVAG_009420;TVAG_241160;  TVAG_009460;TVAG_228520;  TVAG_471530;TVAG_320780;  TVAG_491670 | Malic enzyme | 3.11 | --- |
| TVAG_457850 | teneurin and N-acetylglucosamine-1-phosphodiester alpha-N-acetylglucosamineidase | 3.01 | yes |
| TVAG_154680 | conserved hypothetical protein | 2.74 | --- |
| TVAG_183790 | malic enzyme, putative | 2.71 | --- |
| TVAG_467900 | Conserved Hypothetical Protein | 2.53 | yes |
| TVAG_238830 | malic enzyme, putative | 2.50 | --- |
| TVAG_430830;TVAG_364620;  TVAG_079260 | phosphofructokinase, putative | 2.49 | yes |
| TVAG_371570 | 4-alpha-glucanotransferase, putative | 2.33 | --- |
| TVAG_372530 | serine/threonine-protein kinase ripk4, putative | 2.34 | --- |
| TVAG_487340 | galactokinase, putative | 2.29 | --- |
| TVAG_340570 | Conserved Hypothetical Protein | 2.23 | yes |
| TVAG_573910 | Conserved Hypothetical Protein | 2.22 | yes |
| TVAG_421580 | clathrin coat associated protein ap-50, putative | 2.20 | --- |
| TVAG_338530;TVAG_148390;  TVAG_289290;TVAG_525430;  TVAG_073810;TVAG_034440;  TVAG_200200 | tubulin, putative | 2.16 | --- |
| TVAG_192540 | conserved hypothetical protein | 2.14 | --- |
| TVAG_286280 | conserved hypothetical protein | 2.11 | yes |
| TVAG_249080 | snare proteins, putative | 2.02 | yes |
