# Supplementary material for: A Novel Trichomonas vaginalis Surface Protein Modulates Parasite Attachment via Protein:Host Cell Proteoglycan Interaction
Source: mBio. 2021 Feb 9;12(1):e03374-20. doi: 10.1128/mBio.03374-20 (PMC7885099; doi:10.1128/mBio.03374-20)
Supplement: FIG S5 [file mBio.03374-20-sf005.docx]

Fig. S5
